# Additive Manufactured Parts Produced Using Selective Laser Sintering Technology: Comparison between Porosity of Pure and Blended Polymers

**DOI:** 10.3390/polym15224446

**Published:** 2023-11-17

**Authors:** Chiara Morano, Leonardo Pagnotta

**Affiliations:** Department of Mechanical, Energy and Management Engineering, University of Calabria, 87036 Rende, CS, Italy; chiara.morano@unical.it

**Keywords:** additive manufacturing, 3D printing, selective laser sintering, porosity, pure polymers, blended polymers

## Abstract

For different manufacturing processes, porosity occurs in parts made using selective laser sintering (SLS) technology, representing one of the weakest points of materials produced with these processes. Even though there are different studies involving many polymeric materials employed via SLS, and different manuscripts in the literature that discuss the porosity occurrence in pure or blended polymers, to date, no researcher has reported a systematic and exhaustive comparison of the porosity percentage. A direct comparison of the available data may prove pivotal in advancing our understanding within the field of additively manufactured polymers. This work aims to collect and compare the results obtained by researchers who have studied SLS’s applicability to different amorphous or semi-crystalline polymers and pure or blended materials. In particular, the porosity values obtained by different researchers are compared, and tables are provided that show, for each material, the process parameters and the measured porosity values.

## 1. Introduction

Although the use of metals is constantly growing [1], polymers are still the most used materials today [2] in additive manufacturing (AM) [3].

Currently, most components made with polymeric materials are manufactured using the selective laser sintering (SLS) process [4]. This process belongs to one of the first-born families of AM processes, called Powder Bed Fusion (identified with the acronym PBF), which is based on the fusion of layers of powdered material. The SLS process, in particular, uses thermoplastic polymeric powders, and their fusion is obtained, layer by layer, using a laser beam that acts along directions selected using a computerized system. SLS is one of the most widespread AM processes. In principle, any polymer that is available in powder form, which can be melted and bonded without decomposition via heating, would appear to be processable using selective laser sintering. In practice, however, today, due to the very complicated and difficult-to-control physical phenomena involved in the process [5,6], there are only a few polymers that are suitable for SLS [7,8,9,10]. Both amorphous and semi-crystalline polymers have been studied and employed in SLS processes, with the latter being the most popular [11,12].

In the market of materials that are available for SLS processes, polyamide-based powders 11 (PA11) and 12 (PA12) dominate, followed by other polymeric powders such as BPT, PC, PE, PEBA, PEEK, PET, PMMA, PP, PS, SEBS, TPE, TPU [7], and very few other types. More than 90% of the industrial consumption of polymers for SLS comprises pure Polyamide 12 (PA12) or reinforced blends, such as dry blends of glass-, aluminum-, and carbon-fiber-filled polyamides [10].

The quality of the parts produced using SLS could greatly be affected by the fabrication process, i.e., the powder state, powder particle size, and shape [13,14], and the process parameters [15,16,17]. It is also well known that, as for different manufacturing processes, porosity occurs in parts made using SLS technology. Porosity occurs due to the intrinsic phenomena involved during the melting, sintering, and consolidation processes of powders [18] and represents one of the weakest points of materials produced with these processes. Due to that, the key point behind the widespread use of 3D-printed parts for structural application in different industrial fields is the improvement in product reliability, e.g., defect and porosity reduction. In fact, porosity dramatically affects the quality and reliability of additively manufactured parts and, therefore, deserves great consideration. In recent years, additively manufactured materials have been studied through both numerical and experimental methods to try to understand the effect of porosity (shape, size, number, and position of pores) on critical mechanical properties, such as stiffness, strength, and toughness, and to establish the correlations between these [9,19,20]. Many efforts were made to study how the process parameters affect the porosity level. It was demonstrated that, by optimizing these parameters in the best possible way, the porosity level of the manufactured parts is reduced and becomes dependent only on the type of material used. Overall, the porosity of the parts fabricated with amorphous materials is higher than that of the parts made with semi-crystalline polymers [18].

The common goal of these studies is to mitigate the effects of porosity by developing methodologies that are capable of reducing or, more ambitiously, controlling the generation of pores during the process and introducing post-processing techniques for their elimination.

Evidently, in this context, porosity measurements play a role of primary importance. The scientific literature boasts a large number of articles that study the various polymers that can be processed with SLS, reporting data on their porosity. Measurements of the porosity and density were carried out with different investigation methodologies, passing from traditional measurement techniques to more modern and sophisticated ones [21]. The easiest technique that could be employed for porosity quantification is Archimedes’ method, which allows for porosity evaluation through a density measurement. However, this technique offers some difficulties related to theoretical density knowledge and does not give any information about the pores’ characteristics [21]. The distributions and shapes of the pores can be observed directly using the microscopy analysis technique [22]. The latter, however, has the disadvantages of being a destructive technique and only allowing for the observation of small sections of the sample. Among other techniques for measuring porosity, microcomputed tomography (μ-CT) is certainly the most powerful. This methodology offers the advantage of providing, in addition to the porosity value, the spatial distribution, shape, and size of the pores, in a non-destructive way [23,24]. Its main drawback is the high cost of the equipment.

The purpose of this paper is to collect and compare the porosity percentage measured by researchers on 3D-printed parts. In particular, works that evaluated the applicability of different polymers to SLS were taken into account. Moreover, both amorphous or semi-crystalline as well as pure or blended polymers were considered, even if, as it stands, the highest amount of research is focused on PA12 parts. As assessed before, the importance of having the correct knowledge of porosity in SLS fabricated parts led to the possibility of improving the mechanical properties of 3D-printed parts and, consequently, their reliability.

Despite the intense research carried out and the numerous papers published, to the knowledge of the authors of this work, to date, no investigator has endeavored to juxtapose the assessed levels of porosity. It is believed that a comparison of the available data may be pivotal for augmenting knowledge in the field.

In this paper, after an overview of the various materials processed using SLS, reported in Section 2, the data that are present in the literature relating to the porosity measured on polymers are collected and compared. In particular, in the Section 3, the data related to parts fabricated by polymers belonging to the polyamide family are first discussed, starting from PA12 and moving on to PA6, PA1010, and PA11 and their blends.

Additionally, the porosity measurements of other pure polymers and polymer blends are compared. Tables are provided, which show, for each material, the process parameters and the measured porosity values. Finally, Section 4 is also included for the critical analysis of the main results found in the literature.

## 2. Brief Outline of the SLS Process and Porosity

**Figure 1:**
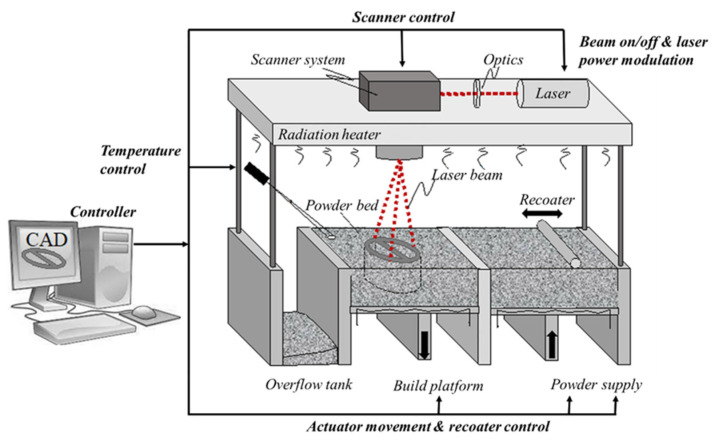
Schematic of an SLS machine’s main components (reprinted from [18]).

A typical system scheme used for 3D printing parts fabricated through SLS technology is shown in Figure 1. For a detailed description of this system, the SLS process, and the influence of the various process parameters on the formation of porosity in the particles produced, please refer to [18].

In short, the manufacturing process involves three stages:

(i) Preheating phase. In this phase, the powder bed is heated to a predefined temperature (bed temperature, T_b_), which is held constant throughout the part-building process. The T_b_ is kept just below the softening temperature of the polymer that is used to minimize the laser energy and eliminate any distortion of the piece during cooling.
(ii) Building phase. This is the core phase of the fabrication process that involves different operations. First of all, the platform is lowered to receive the powder particles dragged by the roller or by the spreading blade. After that, the laser beam melts the layer of particles along the computerized trajectory. Finally, the piece is gradually cooled down to the T_b_ value for solidification.
(iii) Cooling phase. In this phase, the heat source is switched off with the consequent gradual cooling of the powder bed until it reaches the extraction temperature of the piece.

For the numerous parameters involved in the process, refer to Table 1 [18].

In addition to the parameters reported in Table 1, it is important to introduce the Energy Density (*ED*) supplied by the laser to the powder bed. The *ED* stands as an exceptional metric employed by numerous researchers to assess the impact of process parameters on the final part’s quality and porosity. Termed as Andrew’s number, the *ED* quantifies the energy dispensed to particles per unit area of the powder bed surface. Its computation is expressed by the following equation:

(1) ED=P/(v·s)

where *P* represents the laser power (in W), *v* is the scan speed of the laser beam (in mm/s), and *s* is the laser scan spacing, i.e., the distance between two consecutive laser tracks (in mm). The supplied energy density *ED* is then usually given in J/cm^2^, and its value could affect the porosity percentage in the SLS parts. In particular, if the *ED* received by the powder layer is too low or too high, this could lead to an increase in the porosity measured in the parts. Moreover, each of the parameters included in the equation has been found to affect significantly the porosity percentage in the SLS parts [18]. Besides the *ED* parameter, several factors that are not included in the equation could affect the porosity percentage. In particular, among all, the powder bed temperature and the layer thickness are those of the greatest importance. The powder bed temperature influences the cooling rate and viscosity of the polymer during the fabrication process. On the other hand, the layer thickness influences the adhesion characteristics between two consecutive printing layers. To add to these parameters, other factors influence the porosity development mechanisms such as the powder particle sizes, powder re-usage, laser spot diameters, laser scanning strategy, and material properties.

**Figure 2:**
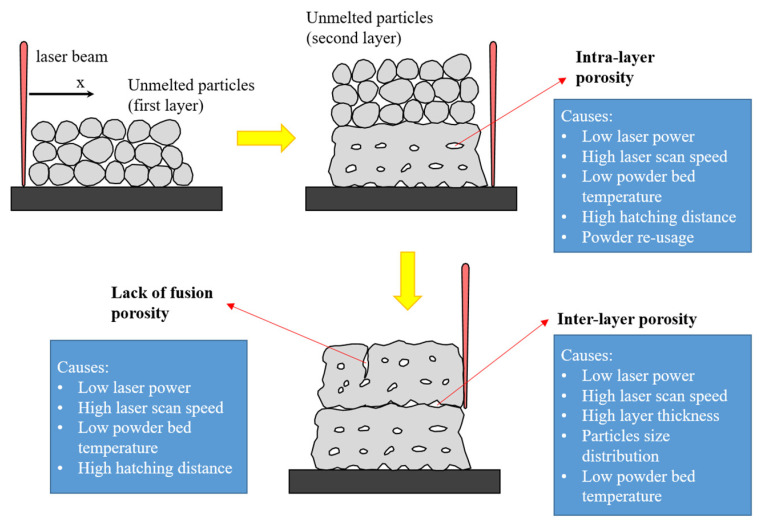
Porosity in SLS parts with respect to processing parameters.

A schematic of the different kinds of porosity that could be found in SLS parts in conjunction with the processing parameters that contribute to their development is reported in Figure 2. In general, porosity is an intrinsic phenomenon of the SLS process. During the melting process, the air could remain entrapped between two adjacent particles, leading to the development of an intra-layer porosity. The amount of these voids is affected by different processing parameters, i.e., the laser power and speed, particle shapes and re-usage, and material properties (viscosity). Beyond that, the porosity may arise due to inconsistent powder deposition as well as an inconsistent energy density received by the deposited powder layer. If the laser power or scan speed is too high or too low, the material layer is too thick, or the hatch distance determining the overlap area and therefore the connection between two hatch lines is too short or too long, this will cause the incomplete melting of the particles by promoting the formation of pores [18,25,26], i.e., a lack of fusion porosity. Finally, porosity could develop between two consecutive layers, i.e., inter-layer porosity. For an updated overview of the nomenclature and measurement methods, refer to [21]. Porosity can be defined through the following ratio:

(2) $$\varepsilon =V_{p}/V$$

where *V* is the part volume and *V*_*p*_ is the pores’ volume. The *V*_*p*_ value could be calculated using different approaches depending on the pore classification. Pore classification could be conducted according to different characteristics. First of all, it is possible to distinguish between open pores and closed pores, in function to the capability to intercept external fluid. A second classification could be conducted based on the pores’ geometry, e.g., cylinders, prisms, spherical cavities, and windows. However, for 3D-printed parts, the occurrence of irregular pores is very high and, consequently, it is not possible to employ this classification method. A third classification is conducted based on the pore size, identified as the smallest pore dimension, i.e., pore width. In this case, it is possible to distinguish between micropores (i.e., pore width < 2 nm), mesopores (i.e., pore width > 2 and <50 nm), and macropores (i.e., pore width > 50 nm) according to the IUPAC classification. However, the one-dimension classification is sometimes not exhaustive, and very often, other 2D and 3D parameters are involved, e.g., areas or volumes. Finally, a fourth classification could be conducted based on the pores’ origins. In this case, it is possible to distinguish between intrinsic pores, i.e., unintentional pores, and extrinsic pores, i.e., pores that are intentionally introduced for a specific application. For a more detailed pore classification, please refer to Morano and Pagnotta’s work [18].

## 3. Polymers for the SLS Process

The SLS process exhibits characteristics that make it suitable for processing different kinds of materials and/or blends. However, thermoplastic polymers (both amorphous and semi-crystalline) are the most widely applied materials in SLS since they require low processing temperatures and, consequently, low laser powers.

A rather comprehensive overview of the SLS polymer powders that are commercially available or reported in the scientific literature has been presented by Tan et al. [10]. The authors classified the different thermoplastic polymers using the pyramidal scheme reported in Figure 2. The first classification is between amorphous (on the left side) and (partially) crystalline (on the right side) polymers. Beyond that, moving upwards, polymers are distinguished based on their mechanical properties, operating temperatures, and costs. In particular, on the bottom, we find the so-called “commodity” polymers, i.e., low-cost polymers for high-consumption applications. In the middle area, it is possible to find the “engineering” polymers, i.e., materials for applications requiring few advanced characteristics, such as moderate temperature resistance and good mechanical properties. Finally, on the top of the pyramid, we find the “high-performance” polymers, i.e., polymers with high costs and high mechanical properties and/or service temperatures. Moreover, the red boxes identify polymers that are commercially available, and the yellow boxes are for polymers that were studied in the laboratory and reported in the scientific literature, while the white boxes are for polymers that are not available for SLS (refer to the abbreviation listed at the end of the paper for the meaning of acronyms).

By analyzing the data reported in Figure 2, it can be seen that, among the thermoplastic polymers available, approximately 25% are not suitable for SLS, 40% are not commercially available even if they have already been tested, while only the remaining part (corresponding to approximately 35%) is currently used in the additive manufacturing industry. It should also be noted that the latter is represented by approximately 85% of semi-crystalline polymers, including polyamides. The latter, even if numerically few by type, are polymers that quantitatively represent almost all of the production of powders. Due to that, these polymers have been and are still the most studied, both in their pure and blended forms.

In the following sections, the polymers of the polyamide family and their compounds will be first discussed. After that, the other semi-crystalline polymers will be considered, starting from the base of the pyramid and ending with the most performing polymer shown on the top of the pyramid. The same methodological sequence will be used to describe amorphous polymers and elastomers.

**Figure 3:**
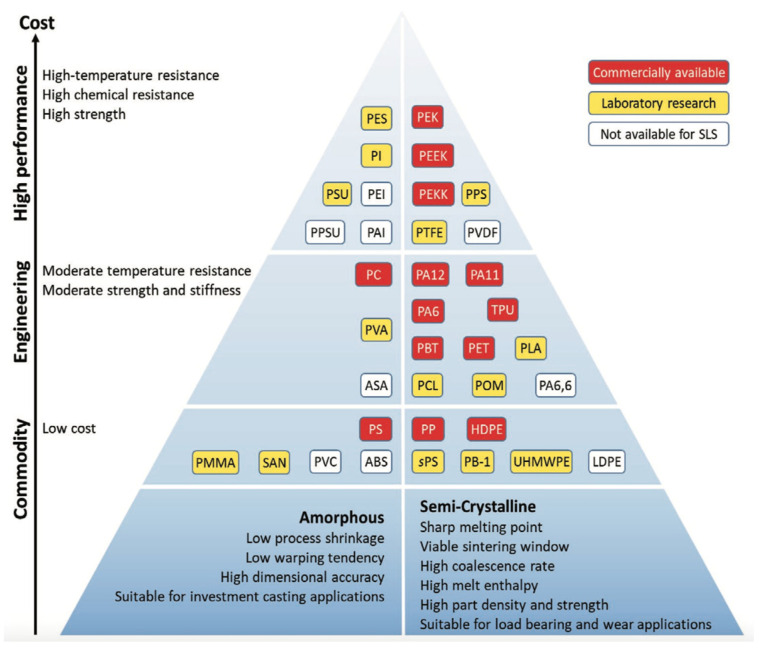
Polymer powders commercially available for SLS. Reprinted from [10].

It should be noted that not all of the polymers that have been investigated up to now are shown in Figure 3. The missing ones, found in the literature by the authors of this paper, will also be reported and discussed, as far as possible.

### 3.1. The Polyamide Family

Polyamide is the most popular polymer in the SLS application since it allows for the creation of parts that have good mechanical properties, good finishes, low costs, and recyclability [27]. In addition, they are also suitable for the production of composite materials and medical applications [28,29].

In addition to the more widespread Polyamide 12, the polyamide family also includes Polyamide 6 (PA6), Polyamide 1010 (PA1010), Polyamide 11 (PA11), and the blends obtained by mixing different polyamide powders. The main results are discussed in the following sections.

#### 3.1.1. Polyamide 12

Actually, polyamide 12 is the most applied and studied material for the SLS process [30,31]. Despite this, PA12 parts are still characterized by the occurrence of fabrication voids and defects. Due to that, achieving low levels of porosity in manufactured parts remains a major challenge, and multiple and accurate studies have been conducted to try to identify and explain the mechanisms of pore formation [18,32]. Several researchers have studied how the SLS processing parameters influence the total contents of pores and their distributions within the polymer parts.

Dupin et al. [33] compared the closed porosity, shapes, dimensions, and positions of pores of SLS parts produced from two different PA12 powders (Duraform and InnovPA) by varying the energy density value. In particular, the authors decided to modify only the laser power value by keeping the other parameters fixed. Seven different laser power levels were selected and, consequently, seven ED values. The porosity characteristics were evaluated using both Archimedes’ principle and X-ray tomography. They found that the quantities of open and closed porosities decrease as the ED increases. These results can be explained by taking into account that increasing the ED induces more particles to melt, so the amount of molten polymer increases too. This molten matter promotes the coalescence process between adjacent particles and therefore enhances the densification of the parts. Overall, it is possible to assess that the energy density has a great impact on the residual porosity. The authors also show that the particle size distribution and the crystallization temperature of the powder material are the key parameters in pore formation. It has been observed that the presence of small particles affects the density of the final part as it promotes the adhesion between the layers producing a lower interlayer porosity. This feature also influences the fusion phase since it affects the coalescence process of the particles. On the other hand, the crystallization temperature affects the porosity during the last stage of the process, i.e., the cooling stage. In fact, lower crystallization temperatures imply an increase in the time that the materials spent in the molten stage, i.e., lower porosity [33].

Tontowi and Childs [34] investigated the effect of the powder bed temperature (ambient build powder surface temperature) on the part density. The effect was evaluated both experimentally and numerically by developing a 2D model. The authors showed that small temperature variations have a marked effect on the part density. This result was observed both numerically and experimentally. In particular, the lower the powder bed temperature, the lower the sintered part density. The effect of the bed temperature could be mitigated by varying the energy density value according to temperature fluctuation.

Gomes et al. [35] analyzed the influence of the dust lap on the quality of the PA12 printed parts using a CT analysis. The authors found an increase in the porosity percentage by increasing the recycling cycles. In particular, a very low porosity percentage was measured for the parts fabricated with virgin powder, i.e., around 1.5%. By increasing the number of printing cycles, this value increased up to 9%. The porosity increase was further accompanied by geometrical errors. Powder recycling is a key point for the SLS process since it allows for the reduction in production costs as well as process waste.

Dewulf et al. [36] investigated the influence of laser power, hatch spacing, and scan speed on porosity development. Each parameter influence was evaluated separately by keeping the other values constant. With this approach, it was possible to obtain samples fabricated with the same ED value but with different processing parameter values. It was shown that an increase in the energy density leads to different porosity contents depending on the varied parameters. Moreover, it was found that by reducing the hatching distance, it was possible to reduce the porosity value. Conversely, the minimum porosity value does not correspond to the maximum laser power or the minimum scanning speed. This research demonstrated that the ED value alone is not enough to predict the microstructure of 3D-printed parts.

Pavan et al. [37] analyzed the part density as a function of both the intra-layer time and energy density values. The intra-layer time, i.e., the time between the scanning of a certain point of the layer and the recoating operation, is responsible for the temperature that is locally reached by the powder during the printing process and, consequently, it could significantly affect the morphology of the 3D-printed parts. The authors revealed that the porosity is significantly affected by the combination of the inter-layer time and ED used during the printing process. Even if it is well known that the ED value is a crucial factor in the part density, the authors demonstrated that the intra-layer time has a similar effect. Ensuring a more uniform inter-layer time during the process would allow for a significant reduction in the variation of the product quality.

Stichel et al. [25,38] presented the results of a Round Robin study involving mechanical tensile tests and a microstructural pore morphology analysis of various samples fabricated using different manufacturing machines. The pore morphologies, as assessed through X-ray computed tomography, were juxtaposed and examined in relation to the process parameters utilized and their resultant mechanical properties. Their investigation revealed that the laser energy input parameters exhibited a limited impact on the porosity, in contrast to the prevailing literature, which suggests that a reduction in porosity can be achieved by increasing the laser energy. Conversely, the process temperature, specifically the powder bed temperature, appeared to exert influence over the pore density, with higher temperatures correlating with lower pore densities.

Rüsenberg et al. [39] investigated the porosity at different regions of SLS PA12 cubes, realized by modifying the laser power value, and evaluated the correlation with the main mechanical properties. The authors demonstrated that a higher part density was obtained with a higher energy density, and the mechanical properties were improved. Moreover, the authors found a skin that appears to be significantly denser compared to the internal region. Similar results were obtained by Ajoku et al. [40], by Rouholamin and Hopkinson [41], and by Morano et al. [42].

Liebrich et al. [43] evaluated the occurrence of porosity on thin-walled structures. The measurements were carried out using X-ray microtomography. The authors proved that the porosity within thin-walled structures produced by SLS strongly depends on the wall thickness as well as on the orientation in the building chamber. Overall, the measured porosity values were significantly lower compared to the overall porosity levels reported for laser-sintered parts of greater dimensions.

Morano et al. [42] analyzed the change in the shape and distribution of the pores during quasi-static loading conditions, inducing plastic strain, by employing X-ray micro tomography. The authors found a significant variation in the porosity percentage by increasing the residual deformation. This result was accompanied by a variation of pore shapes and dimensions. The analysis made it possible to follow the main mechanism that contributes to sample failure, e.g., pores’ coalescence.

#### 3.1.2. Porosity and Pore Size Distribution of PA12

The porosity values of PA, measured over the past decade by some of the researchers cited in the previous section, are summarized in Table 2. Alongside the porosity percentage ranges, the table also provides the ranges of values of the process parameters used by various authors to produce the analyzed parts. The measurements were taken at different times and places on parts made with different process parameters and, in some cases, using different techniques. Nonetheless, important considerations can be drawn from their analysis.

First of all, it can be verified that all of the percentage porosities measured are included in the wide range from 0.7% to 16%. The differences between these values can be mainly attributable to the processing parameters used for SLS printing. The latter, as discussed in the previous section, has a strong impact on the structure and on the distribution of pores inside of a finished product and, consequentially, on its mechanical properties [44,45].

However, it should be noted that the maximum values of porosity, reported by Dupin et al. [33], were obtained at lower energy density values, while the minimum values, measured by Liebric et al. [43], were obtained for the case of thin-walled structures. If these particular data are not considered, the variability of porosity can be considered restricted to the range of 2.5–4.8%.

Another important consideration is that the porosity measurements reported by various researchers confirm the correlation between the ED value end porosity percentage, as already observed by Caulfield et al. [26] in 2007. The increase in the ED value, almost always, leads to an increase in the density of the material (or, equivalently, a decrease in its porosity). This correlation could be demonstrated by individually plotting the values provided by various authors in their papers. For the sake of brevity, these data are not all reported in this work. However, trends can be verified by analyzing the data summarized in Table 2, which, for each author, reports only the extremes of the variation intervals. Note that, for each group of data, the ED values increase from left to right, while, on the contrary, the porosity values decrease.

It should be noted that it is not possible to observe a direct correspondence between the ED value and the measured porosity. That could be explained considering that the ED value depends on different factors, i.e., the laser power, hatching distance, and scanning speed (see Equation (1)) [25,46]. Furthermore, porosity also depends on all of the other processing parameters (e.g., bed temperature, layer thickness, etc.; see Table 1), as well as on the measurement method employed for its quantification.

**Figure 4:**
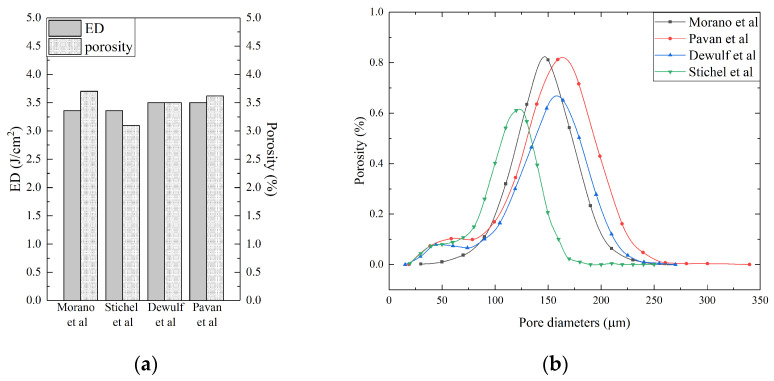
Comparison between (**a**) porosity and (**b**) equivalent pore diameter distribution available in the literature [25,36,42,47].

As an example, Figure 4a reports a comparison between the porosity values obtained by various authors (Stichel et al. [25], Dewulf et al. [36], Morano et al. [42], and Pavan et al. [47]) with approximately equal ED values (ED = 3.4 ± 0.1 J/cm^2^), while, in Figure 4b, the contribution of pores with a specific diameter on the total porosity can be observed. The average value of the measured porosity is equal to 3.5 ± 0.3%. Note that the differences between the measured porosity values are not directly related to the changes in the ED. They are probably attributable to the different process parameters used.

Another general conclusion is that, in all cases, the pore diameter distributions are slightly different. The greatest contribution is given by the pores with an average size that is typically contained between 120 μm and 180 μm. The remaining pores of smaller or larger dimensions, while contributing in a limited way to the overall porosity, can have a great influence on the mechanical properties of the material.

Returning to the correlation between the ED and porosity, it is important to highlight that Erdal et al. [48], as well as Rouholamin et al. [41] and Stichel et al. [25], reported the existence of a maximum optimal energy density. In fact, the authors found that by increasing the energy density beyond this maximum, the level of porosity of the part can remain unchanged or even deteriorate because of thermal degradation.

The sources of variability mentioned above uniquely affect the porosity values obtained by each researcher. This, unfortunately, does not allow for a direct comparison of all the data that are available in the literature in order to extract more general information.

**Figure 5:**
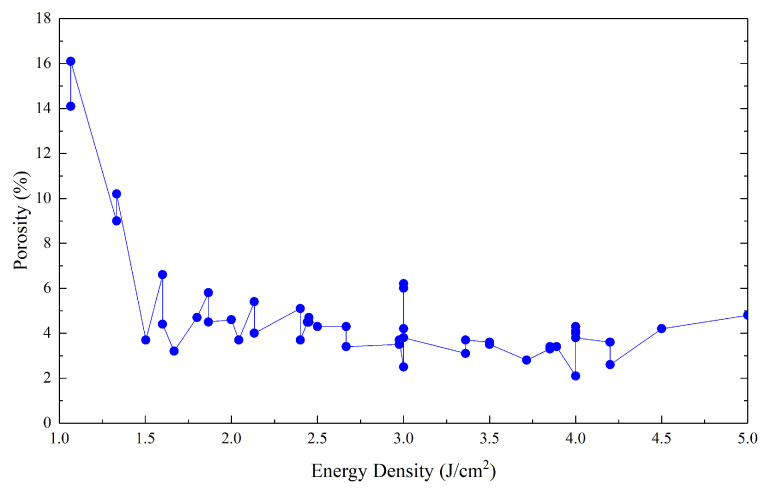
Porosity as a function of energy density. The graph was obtained by grouping all the data available in the literature for PA12.

To confirm this, Figure 5 shows the curve obtained using all the data available in the literature (approximately fifty porosity values measured for different ED levels).

The average porosity obtained is 4.6%, with a large standard deviation of approximately 2.6%. However, when the data are filtered by eliminating the most unlikely values, the average value drops to approximately 4.0% with a standard deviation of 0.94%. However, these values are very far from those presented previously.

**Table 2:** Summary of measured PA12 porosity values available in the literature.

Material	Powder Size (μm)	Layer Thickness (μm)	Powder Bed Temperature °C	Energy Density J/cm^2^	Porosity %	Measurement Technique
PA2200 [25]	60	100–150	160–178	1.67–3.72	3.20–2.80	μ-CT
PA2200 [36]	60	120	-	2.44–4.20	4.70–2.60	μ-CT
PA2200 [42]	56	100	168	3.36	3.70	μ-CT
PA2200 [43]	56	100	-	-	2.60–0.70	μ-CT
PA2200 [47]	56	120	-	2.00–5.00	4.80–3.60	μ-CT
PA2200 [46]	60–80	–	173	3.00–4.00	6.50–2.50	μ-CT
Duraform [25]	58	101–120	165.5–182	1.50–2.04	3.80–3.60	μ-CT
Duraform [33]	60	100	150	1.07–2.67	16.10–4.30	Archimedes μ-CT
Duraform [49]	58	100	175	1.80	4.70	μ-CT
InnovPA [33]	43	100	150	1.07–2.67	14.10–3.40	Archimedes μ-CT

It is difficult to draw other conclusions from the data that are available in the literature on the porosity of PA12. Generally, each researcher has developed their studies by keeping some parameters constant (very often without indicating their values in their published papers) and varying only those of interest, and, except for Stichel et al. [25], everyone used their equipment. It is therefore impossible to try to determine any correlations with the degree of porosity from the published data to understand what method to use to further decrease the porosity level.

It would be desirable for researchers to follow a single direction not only thematically but also for the presentation of the results. Everyone could thus proceed independently and obtain and present results that could be useful to the entire scientific community that studies the porosity of polymers. Currently, many works deal with the influence of the ED, but few investigate the influences of other parameters, so researchers should work in this direction in the future.

### 3.2. Polyamide 6 (PA6), 1010 (PA1010), and 11 (PA11) and Polyamide Blends

Despite the commercial availability of different polyamide powders for SLS printing, the literature on polymer provides, in general, only little information on their processing, since the vast majority of published results are focused on PA12.

Nevertheless, the data in the literature about the porosity percentage measured on SLS parts fabricated using different polyamide powders are summarized in Table 3.

One of the other polyamide powders available for 3D printing is PA6. Zhou et al. [50] investigated Polyamide 6 single-layer specimens. The hatch spacing and the processing temperature were varied to evaluate their influences on the sample characteristics and porosity development. The authors demonstrated that the hatch spacing significantly affects the occurrence of layer porosity. In particular, the porosity ratio increases drastically as the scan spaces enlarge, passing from 1.95% to 3.89% when the scanning space increases from 0.25 to 0.45 mm, with an increment of about 50%. On the other hand, the processing temperature affects the mechanical properties.

Ling et al. [51], instead, analyzed, among other things, the porosity ratio of sintered specimens with different ambient temperatures and layer thicknesses. The experimental results demonstrated a decrease in the porosity ratio by increasing the processing temperature, i.e., from 60% measured at 25 °C to approximately 39% measured for temperatures up to 180 °C. These measured values are significantly higher than those reported by Zhou et al. [50]. This difference could be attributed to different printing parameters and sample characteristics. In particular, such high porosity values were measured on one-layer samples. This aspect demonstrated that the layer-by-layer process helps to reduce porosity since some void could be closed during the second layer melting. In fact, the porosity percentage measured on the samples realized with different layers, from 3 to 10, showed a decrease of up to 21% by increasing the number of layers. By further increasing the number of layers, the porosity decrease was slow, and for a number of layers that exceeded 14, it was negligible.

Another commercial powder that is available is PA1010. Liu-Ian et al. [52] investigated the morphology changes of a modified PA1010 by varying different processing parameters, i.e., the laser power, powder bed temperature, and layer thickness. The author found that by increasing the laser power, i.e., by more than 8 W, it is possible to obtain a well-defined morphology. However, for a laser power greater than 15 W, polymer degradation was observed. Similarly, increasing the bed temperature allows for a reduction in the dimensions of the detected pores. Finally, by reducing the layer thickness, it is possible to improve the sample morphology, i.e., lower the porosity, even if, for a thickness lower than 0.05 mm, the roller compromises the sample surface. Overall, even if authors analyze the porosity morphology, they do not quantify the amount.

Finally, PA11 has seen increased interest in general use due to its sustainable nature, since it is unique among other polyamides, as it is non-petroleum sourced. To fill the gap left by the literature on how to achieve optimal processing conditions, Wegner et al. [53] studied the correlations between the process parameters and part properties using a Design Of Experiments (DOE) approach. In particular, the main processing parameters, i.e., the scan speed, the laser power, hatch distance, and layer thickness, were modified, and their influence on the sample characteristics was analyzed. In particular, the authors evaluated the part density, surface roughness, and the final mechanical properties. Also, in this case, the authors did not furnish data about the porosity percentage. However, the experimental results demonstrated that energy density values that are significantly higher than those employed for PA12 fabrication are requested to achieve dense parts.

For the sake of completeness, this section closes by highlighting that, recently, some studies have been carried out to verify the possibility of using mixtures of polyamide powders in the SLS process. In particular, the works of Salmoria et al. [54] and Strobbe et al. [55] examined the properties of PA12 blends with PA6 and PA4,6 powders, respectively.

### 3.3. Other Pure Polymers and Polymer Blends

Among the AM techniques, SLS gives the possibility to process a wider range of polymeric powders [11], including a variety of pure-polymer-based powders. Comprehensive reviews on materials and process development are given by Kruth et al. [56], Schmid et al. [6,57], and, more recently, by Tan et al. [10]. Schmid et al. [57], in particular, discussed why several approaches adopted for new types of polymers failed and the reasons for the difficulties in developing new SLS powders.

Typically, polymer powders that are employed for the SLS process are semi-crystalline thermoplastic, even if it is also possible to find amorphous polymeric powder as well as elastomers. Thermoplastic polymer materials are well suited for laser sintering because of their relatively low melting temperatures.

In fact, if we exclude the polyamide varieties that were already examined in the previous section, a very limited variety of other kinds of polymers has been the subject of scientific publication. In this section, studies in which the porosity has been investigated are discussed, and the main results are summarized in Table 4.

Schmid et al. [58,59] presented a process chain for the production of spherical polybutylene terephthalate (PBT) microparticles. Their PBT powder, having a melting point of 223 °C, could be processed using a building temperature of 210 °C. Overall, by carrying out a rounding and a drying process on powder particles, it was possible to obtain a material suitable for 3D printing. However, further optimization is needed to improve the density of bulk parts.

Arai et al. [22] proposed to use a copolymer PBT (cPBT) for the fabrication of polymeric powder for the SLS process. The authors employed a cryomilling process for powder fabrication. It was found that the employed methods led to the occurrence of some metallic particle contaminations. These particles are responsible for an increased crystallization temperature that reduces the process windows. Nevertheless, the so-obtained cPBT powder was successfully employed for part fabrication with the SLS process. Table 4 summarizes the results in terms of porosity for different layer thicknesses and energy densities.

**Table 4:** Summary of measured porosity values available in the literature for different polymers or blends.

Material	Powder Size (μm)	Layer Thickness (μm)	Powder Bed Temperature °C	Energy Density J/cm^2^	Porosity %	Measurement Technique
PBT [58]	25	295–450	210	8.4–12.6	6.3–14.1	Archimedes
PBT [22]	76	100	190–193	6.7–40	1.7–20.8	Micrographs
HDPE [60]	150–212	200	95	44	35	Archimedes
UHMPE [61]	125	100	142	1.6–3.2	60–65	Archimedes and μ-CT
PP [9]	45	150	150	1.8–1.9	8.4–10.1	μ-CT
PET [62]	59	100	200–240	2–5	2	Micrographs
PEEK 150PF [63]	56	100–200	345–357	1–3.6	0.2–15	Archimedes
PEEK 450PF [64]	50	120	-	1.47–3.24	0.35–17	μ-CT
PEEK HP3 [65]	60	100	-	-	4.36	Mercury intrusion
PEK HP3 [66]	70	120	368	-	0.3–10.4	μ-CT
PEK HP3 [67]	37–63	120	340	-	-	-
POM [68]	87–146	200	154–159	-	-	-
BLENDS						
PA12/PEEK [69]	80	100	-	4.5	-	-
SEBS/PP [70]	85–107	100	100–160	7.9	-	-
PBT/PC [71]	161/218	-	205	5–10	10–40	Micrographs
PP/PA12 [72]	-	100	158	-	-	-
PA12/PBT [73]	60/200	-	140	38	-	-
PA12/HDPE [74]	60/120	150	60	-	-	-

Other semi-crystalline polymers such as polyethylene, polypropylene, polyoxymethylene, poly(ether ketone), and poly(ether ether ketone) are being actively researched, and some have been commercialized [7].

Bai et al. [75] explored, for the first time, the processability of polyethylene via selective laser sintering. The authors evaluated the influence of the processing parameters, e.g., the powder bed temperature, and laser power on the mechanical properties of 3D-printed parts. Moreover, the effect of the thermal history during the laser sintering process has also been evaluated. Unfortunately, the authors did not report any data about the porosity but evaluated only the quality of the fabricated parts.

Salmoria et al. [60] investigated the fabrication of HDPE specimens via SLS, employing particles with different sizes to control the porosity variation. The authors showed that the pore dimension depends on the sintering degree as well as on the particle size. In particular, the dimension of closed pores increases by increasing the dimension of the particles employed for 3D printing.

Khali et al. [61] carried out a mechanical and morphological characterization of porous Ultra-High-Molecular-Weight Polyethylene (UHMWPE) laser-sintered samples realized by using different processing parameters. Different works in the literature demonstrate that is difficult to fabricate UHMWPE parts via additive manufacturing. Due to that, the authors evaluated the influence of laser power variation on 3D-printed UHMWPE parts. The results demonstrated that the porosity level remains high (ranging between 60% and 65%) with no significant variation by modifying the laser power value and, consequently, the flexural properties are compromised.

Poly(ethylene terephthalate) (PET) was found to be suitable for application in SLS. Bashir et al. [62] analyzed the feasibility of processing highly crystalline PET for SLS 3D printing. It was found that the material exhibits a wide operating window and the recyclability of unmelted powder for new cycles is good. Overall, it seems that the printability of PET is similar to that of PA12. Moreover, the authors measured a 2% residual porosity.

In recent years, high interest was given to a new type of polymers that are suitable for high temperatures, i.e., Poly Aryl Ether Ketones (PAEKs), for the SLS process. Examples are Poly Ether Ketone (PEK) and Poly Ether Ether Ketone (PEEK), which could be successfully employed in different industrial fields, thanks to their high melting temperatures, chemical and wear resistance, and biocompatibility [63]. Even if PA and PS polymer families are widespread in different industrial processes, the processability of PEEK through 3D printing is currently a challenge [66]. However, it is necessary to include some printer variation to increase the process temperature up to 350 °C, i.e., the melting temperature of this kind of polymer. Moreover, it is also necessary to improve the materials’ flowability. It was also shown that it is possible to reduce the porosity from 15% to a nearly zero value by properly selecting the process parameters. In this way, it is also possible to improve the mechanical properties that appear to be interconnected to the porosity percentage. In the literature, different studies were carried out on the feasibility of PEK, PEEK, and EOS PEEK HP3 parts using the SLS process [64,65,67,76].

The SLS technology could be employed for PP powder processing. However, it is necessary to deeply analyze the process parameters’ influence on PP 3D-printed parts for reliable manufacturing. Flores Ituarte et al. [9] evaluated the influence of the main processing parameters’ variation on the porosity percentage. In particular, a DOE was developed to investigate the influence of both the laser power and scanning speed. The porosity was measured through computed tomography. It was found that the occurrence of a high porous structure, i.e., a porosity percentage ranging between 8.46% and 10.08%, and, moreover, the highest porosity appears to be located in the interlayer planes.

Other polymers that exhibit good mechanical properties such as high stiffness, high wear, and creep resistance are PBT and POM. However, studies on this kind of polymer are scarce. Recently, Wegner [76] analyzed the percentage of bulk density of different polymeric parts fabricated through two different laser sintering machines. A porosity percentage between 1% and 2% was found, which is lower than the typical values measured on PA 12. Dechet et al. [68] employed a non-mechanical method, based on the solution–dissolution process, for the fabrication of POM powders that are suitable for PBF. The quality of the as-manufactured powder was demonstrated through the manufacturing of multi-layered samples.

Finally, polymer blends were developed and analyzed for the fabrication of parts with improved properties. This kind of material offers an alternative approach for obtaining parts with specific characteristics, thus allowing for the development of new applications. Nonetheless, polymer blends have received considerably less attention in research compared to pure polymers. This disparity arises from the necessity for chemical compatibility between the constituent materials in the blend and the thermal limitations that make the sintering of such blends more challenging. Additionally, the temperature ranges within which the sintering process must occur tend to be narrower for polymer blends than for their pure polymer constituents. This implies that polymer blends are more susceptible to variations in the bed temperature of the part, underscoring the critical importance of precise temperature control. The utilization of SLS for polymer blends is contingent upon a broad selection of compatible blend constituents. Nevertheless, there have been noteworthy developments in the application of SLS to various polymer blends including PA12/PEEK [69], PA12/HDPE [74], PA12/PBT [73], PA12/PP [72], PBT/PC [71], PMMA/PS [77], PP/POM [76], and SEBS/PP [70]. The data are reported in Table 4.

### 3.4. Amorphous Polymers and Elastomers

Amorphous polymers were the first kind of polymers employed for SLS. The main results regarding amorphous polymers and elastomers are summarized in Table 5.

Among amorphous polymers, polycarbonate is widespread, and it includes bisphenol-A PC and aliphatic PC. Bisphenol-A PC exhibits good mechanical properties, and due to that, it is possible to find different studies [78,86,87]. However, bisphenol-A is classified as a low-poison chemical material, and due to that, in different countries, its use is forbidden for applications in food and medical fields. Consequently, aliphatic PC is subjected to increasing interest. The influence of the main processing parameters and, in particular, of the laser power energy on the aliphatic polycarbonate porosity was analyzed by Song et al. [87]. The experimental results show the occurrence of high porosity, between 50% and 70%. Overall, the process allows for the feasibility of the fabrication of aliphatic PC samples via 3D printing, even if it is still necessary to reduce the porosity.

As regards polystyrene (PS) and high-impact polystyrene (HIPS), the research in this field is limited. Only very few works have been published in the open literature [79,80,81]. For example, Shi et al. [80] evaluated the printability of high-impact polystyrene and found good dimensional accuracy as well as mechanical properties. Similarly, Strobbe et al. [81] analyzed the same material using a single-layer approach and evaluated the printing parameters’ influence. A good consolidation of 3D-printed parts and a small amount of porosity was found by properly selecting 3D printing parameters.

Polymethylmethacrylate (PMMA), a synthetic resin [77,82], is employed in medical fields. Typically, this material is mixed with other additives to obtain a soft substrate that can harden gradually. In fact, this material is processed using two different approaches: (i) molding, which is employed when the PMMA is in the soft condition, or (ii) machining after the reaching of the hard form. However, these technologies are accompanied by some limitations such as the possibility to fabricate complex parts. Additive manufacturing could overcome this issue. Velu et al. [82] evaluated the influence of processing parameters on PMMA parts fabricated through SLS. The authors found a correlation between porosity and processing parameters as well as a correlation to mechanical properties. In particular, through the proper selection of process parameters, it is possible to reduce the porosity (52% instead of 61%) and to improve the mechanical properties (tensile strength is two times higher).

As regards the elastomers, among them, one of the most used for SLS is TPU, even if its applicability fields are limited. Currently, there are limited studies on the correlation between the processing parameters and TPU sample quality, e.g., porosity occurrence. Verbelen et al. [83] carried out an experimental analysis of different TPU grades characterized by very distinct characteristics. The authors demonstrated that it is possible to employ the SLS process for the fabrication of TPU parts. However, the final parts are characterized by high porosity and degradation. Due to that, further studies are requested. Ziegelmeier et al. [84,88] carried out different studies on TPU processability using SLS. Firstly, the authors evaluated the correlation between the behavior of the powder and the final properties of the 3D-printed parts. With this aim, the authors selected two different kinds of elastomers, i.e., TPU and a commercial thermoplastic elastomer (i.e., the Duraform Flex-DF). The authors demonstrated that improved packing and flow capability of the powder particles could lead to bulk parts characterized by lower porosity.

Yan et al. [85] studied a styrene–acrylonitrile copolymer (SAN), another kind of amorphous polymer, as an SLS material to make parts with good dimensional accuracy and sintering properties and, consequently, better mechanical properties. In particular, the authors evaluated the influence of processing parameters, e.g., the ED value, on the quality of 3D-printed parts, e.g., the porosity and dimensional accuracy. Moreover, the results were compared with the data on the parts fabricated using PS powder. It was found that SAN could be successfully employed for the fabrication of parts with complex shapes and good dimensional accuracy. However, a high occurrence of voids was detected, and that issue was overcome by employing a post-processing treatment, i.e., infiltrating epoxy.

## 4. Discussion

**Figure 6:**
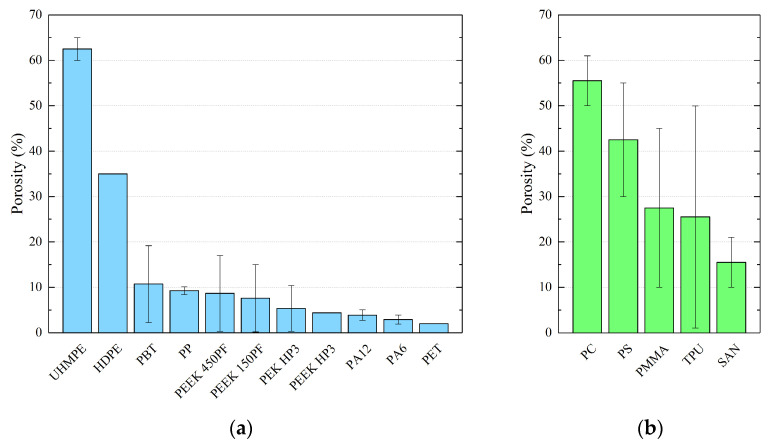
Porosity average value found in the literature for (**a**) semi-crystalline polymers and (**b**) amorphous polymers and elastomers.

For the convenience of treatment, all of the porosity values found in the literature are summarized in the graphs of Figure 6. In particular, Figure 6a reports the data relating to semi-crystalline polymers, while Figure 6b shows the porosities of the amorphous polymers. The values presented are the average of the data reported in Table 2, Table 3, Table 4 and Table 5, neglecting out-of-range values and blends.

The comparison between the porosities of semi-crystalline polymers (Figure 6a) and the porosities of amorphous polymers and elastomers (Figure 6b) clearly confirms what has already been highlighted previously. Semi-crystalline polymers are characterized by lower porosity values. Except for UHMPE and HDPE, which represent two particular cases, the ratio between the porosity values of the two polymer families is greater than 5/1. UHMPE and HDPE are not generally used for the SLS process, and their printability is still a challenge. Porosity values will certainly reduce drastically in the future. Excluding these two polymers, the porosity in semi-crystalline polymers remains less than 10%, and it should be highlighted that an appropriate choice of processing parameters could allow for values close to zero to be reached. The porosities of amorphous and elastomeric polymers are, however, higher and vary between 15% and 55%.

By considering each polymer family, the first outcome that emerged from the literature analysis is that the polyamide family represents the highest percentage of the polymers employed for SLS. In particular, PA12 is a widespread material, and several works have been carried out on this material. The experimental evaluation of porosity on that material demonstrates a high variability, from a minimum of 2.5% to a maximum of 17% [33,36,46]. These values were measured on the samples that were obtained with different processing parameters, thus demonstrating that the fabrication process significantly affects the morphology of 3D-printed parts. Overall, a reduction in the measured porosity was observed by increasing the ED value. Higher supplied energies give the possibility of increasing the dimension of the melting pool and reducing the material viscosity, thus obtaining a denser part. However, it is not possible to limit the correlation of the porosity value to the ED value. In fact, in some cases, with similar ED values, different porosity values were measured (see Figure 4 and Table 2). These discrepancies could be attributed to the other processing parameters that are not involved in the ED equation. Moreover, the same ED values could be obtained, starting from different processing parameters that could influence the quality of 3D-printed parts in different ways [36]. Two main considerations follow from these results. First of all, it is clear that it is possible to reduce the porosity by acting on fabrication parameters; secondly, the complete elimination of the porosity could not be obtained just by acting on process parameters. Moreover, it is also important to consider that different techniques employed for porosity evaluation could lead to different measuring errors, thus contributing to the variability. All of these considerations suggest the need to develop standardized strategies and protocols for the fabrication of 3D-printed parts and their quality assessment.

Similar considerations could be made on the other polyamide raw powders as well as for the other polymers or blends. However, for these materials, the data in the literature about porosity are limited and reveal a very high variability (see Table 3, Table 4 and Table 5). Moreover, in some cases, the information about fabrication parameters does not give the possibility to make specific comparisons between different analyses carried out by different researchers. Overall, the higher porosity values observed for some SLS polymers could be attributed to two main factors. On one hand, some of the considered materials have been employed in the SLS process in the past few years. Due to that, the process is still under development, and further optimization is needed. On the other hand, some of these materials are not suitable for traditional SLS processes, such as those materials that require high melting temperatures. In this case, the SLS process and machines should be optimized for this kind of material. Overall, it is possible to conclude that porosity is still an open issue for SLS-printed polymers and that further analysis is required, especially for new emerging polymers with high mechanical properties, e.g., PEEK.

## 5. Conclusions and Future Remarks

In this review paper, a wide description of the main polymeric materials employed for the SLS process is reported. An analysis of the literature demonstrated that the widespread material for SLS is PA12. However, the analysis of the process parameters and their influences on the mechanical properties and part density is actually a crucial aspect, and different researchers are focusing their efforts on investigating this point. In fact, a deep understanding of this correlation is a key point for improving the reliability of SLS parts. The experimental results obtained by many researchers on PA12 allowed us to observe a correlation between the ED value and the porosity percentage that decreases by increasing the ED. However, the ED value alone is not enough to predict the porosity percentage in 3D-printed parts, since other processing parameters significantly affect the porosity development, e.g., the powder bed temperature or layer thickness. It is also important to highlight that the porosity values refer only to the percentage of porosity without giving information about the pores’ shapes. However, it was demonstrated that the pores’ dimensions and shapes could affect the mechanical properties as well as the porosity percentage itself. For this reason, further investigation is needed to analyze the influence of the processing parameters on the pores’ geometrical characteristics.

An analysis of the literature also demonstrated the increasing research around other kinds of polymers for structural application, e.g., PEEK, or medical application, e.g., PMMA. The data about porosity are reported in specific tables, and the results demonstrated that the high product quality of 3D-printed parts is still an open issue, and further investigation is requested. Overall, the way to achieve the deployment of SLS technology in different industrial fields lies in the possibility of developing standard strategies for part fabrication and, as a final goal, fabrication protocols.

## Figures and Tables

**Table 1:** SLS process parameters (reprinted from [18]).

SLS Process Parameters			
Powder-Based	Laser-Based	Temperature-Based	Scan-Based
Particle shape, size, number, and spatial distribution	Laser power	Powder bed temperature	Scan speed
Powder flowability	Spot size	Powder feeder temperature	Hatching distance
Recoating speed, layer thickness, and powder density	Pulse duration	Temperature distribution	Scanning pattern
Material Properties	Pulse frequency		

**Table 3:** Summary of measured porosity values available in the literature for different polyamide parts.

Material	Powder Size (μm)	Layer Thickness (μm)	Powder Bed Temperature °C	Energy Density J/cm^2^	Porosity %	Measurement Technique
PA6 [50]	180	500	25–150	-	1.9–3.9	Micrographs
PA6 [51]	100	100	25–180	2–36.6	39.7–60	Archimedes
PA1010 [52]	20–110	100–250	90–102	-	-	-
PA11 [53]	-	80–120	187	1.83–5.40	-	-
PA6/PA12 [54]	150–160	150	120	-	-	-
PA4,6/PA12 10/90 [55]	-	100	162	2.5–8	3.5–4.5	Archimedes
PA4,6/PA12 50/50 [55]	-	100	162	2.5–8	4–5	Archimedes
PA4,6/PA12 90/10 [55]	-	100	162	2.5–8	4.5–6.5	Archimedes

**Table 5:** Summary of measured porosity values available in the literature for different amorphous polymers or elastomers.

Material	Powder Size (μm)	Layer Thickness (μm)	Powder Bed Temperature °C	Energy Density J/cm^2^	Porosity %	Measurement Technique
PC [78]	30–180	130	145	3–12	10–45	Archimedes
PS [79]	25–106	150	85	2–12	12–60	Archimedes
PS [80]	75–100	150	90–95	6–7	-	-
PS [81]	-	100	90–100	4–14	5–25	Archimedes
PMMA [82]	75	-	100	15–40	50–61	Archimedes
TPU [83]	63–75	100	70–125	5–14	10–21	Archimedes
TPU [84]	45.7–62.8	100	125	25	0–0.2	Archimedes and μ-CT
SAN [85]	59.08	100	99	2–12	30–55	Archimedes

## Data Availability

The data reported in this work are available upon request.

## Abbreviations

ABS	Acrylonitrile Butadiene Styrene
AM	Additive Manufacturing
ASA	Acrylonitrile Styrene Acrylate
ASTM	American Society for Testing and Materials
CAD	Computer-Aided Design
CT	Computed Tomography
DOE	Design Of Experiments
HDPE	High-Density Polyethylene
PA	PolyAmide
PAEK	PolyArylEtherKetones
PAI	PolyAmideImide
PBF	Powder Bed Fusion
PB	PolyButylene
PBT	PolyButylene Terephthalate
PC	PolyCarbonate
PCL	PolyCaproLactone
PEEK	PolyEtherEtherKetone
PEI	PolyEtherImide;
PES	PolyEtherSulfone
PET	PolyEthylene Terephthalate
PI	PolyImide
PLA	PolyLacticAcid
PMMA	Poly(Methyl MethAcrylate)
POM	PolyOxyMethylene
PP	PolyPropylene
PPF	Poly(PropyleneFumarate)
PPSF	PolyPhenyl Sulfone
PPSU	Poly(Phenyl Sulfone)
PS	PolyStyrene
PSU	PolySulfone
PTFE	PolyTetraFluoroEthylene
PU	PolyUrethane
PVA	Poly(VinylAlcohol)
PVAc	PolyVinylAcetate
PVB	PolyVinylButyral
PVC	Poly(VinylChloride)
PVDF	PolyVinyliDene Fluoride
RP	Rapid Prototyping
SAN	Styrene-AcryloNitrile
SLS	Selective Laser Sintering
sPS	Syndiotactic PolyStyrene
TPE	ThermoPlastic Elastomers
TPO	Phosphineoxide
TPU	Thermoplastic PolyUrethane elastomer
UHMWPE	Ultra-High-Molecular-Weight PolyEthylene
